# Biomarkers of Trifluridine-Tipiracil Efficacy

**DOI:** 10.3390/jcm10235568

**Published:** 2021-11-26

**Authors:** Ioannis A. Voutsadakis

**Affiliations:** 1 Algoma District Cancer Program, Sault Area Hospital, Sault Ste. Marie, ON P6B 0A8, Canada; ivoutsadakis@yahoo.com or ivoutsadakis@nosm.ca; 2 Section of Internal Medicine, Division of Clinical Sciences, Northern Ontario School of Medicine, Sudbury, ON P3E 2C6, Canada

**Keywords:** TAS-102, biomarkers, predictive, gastrointestinal cancers, colorectal

## Abstract

Trifluridine/tipiracil (TAS-102) is a newer generation chemotherapy that has been approved for the later-line treatment of metastatic colorectal and gastric/gastroesophageal adenocarcinomas. The oral drug provides a modest benefit of prolongation of survival over placebo in pretreated patients with these cancers with acceptable toxicity. Studies have shown rare objective responses (2–4%), and the disease control rates were 44% in both colorectal and gastric cancer randomized trials. Thus, the majority of patients progress through treatment and are burdened by toxicities. To better characterize the sub-group of patients with a higher probability of benefit from trifluridine/tipiracil, predictive biomarkers have been sought using data from randomized trials as well as from non-randomized trials and real-world series. Biomarkers examined include clinical characteristics of the patients, laboratory tests, and tumor derived biomarkers. These studies show that early neutropenia on treatment, and ratios of leukocyte subsets, are potential biomarkers able to predict trifluridine/tipiracil benefit. Combinations of laboratory values and clinical characteristics and proteins involved in trifluridine transport and activation have been examined with initial positive results.

## 1. Introduction

**Figure 1:**
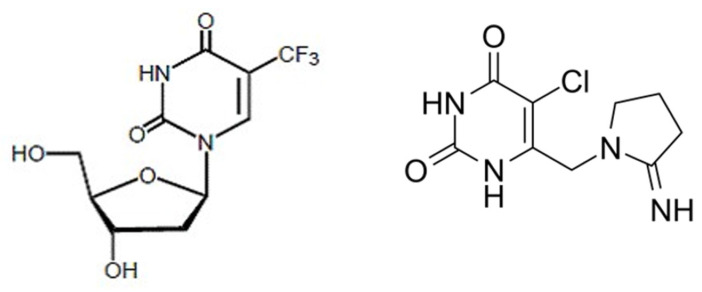
Chemical structures of the components of TAS 102: trifluridine on the left and tipiracil on the right.

Gastrointestinal cancers cause significant morbidity and mortality worldwide. Colorectal cancer, in particular, is the most common gastrointestinal cancer and is responsible for more than 52,000 deaths annually in the United States of America alone [1]. Despite improvements in therapy, metastatic colorectal cancer is curable only in a minority of oligometastatic cases. Metastatic disease that has become refractory to chemotherapy with fluoropyrimidine-based combinations has a dismal prognosis and few therapeutic options [2]. One such option introduced in recent years is TAS-102, a fixed 1 to 0.5 ratio dose combination of the antimetabolite α,α,α-trifluorothymidine (trifluridine) with tipiracil (5-chloro-6-(2-iminopyrrolidin-1-yl)methyl-2,4(1,3)-pyrimidinedione hydrochloride), a thymidine phosphorylase inhibitor (Figure 1) [3,4]. Trifluridine/tipiracil has been approved for the treatment of metastatic colorectal cancer patients who have previously received fluoropyrimidine, oxaliplatin, irinotecan, anti-VEGF targeted therapy and anti-EGFR targeted therapy, if the *KRAS* wildtype gene is detected [5]. The basis of approval was the RECOURSE randomized phase 3 trial (NCT01607957) that showed prolongation in both overall survival (OS) and progression-free survival (PFS) with trifluridine/tipiracil versus placebo, with acceptable toxicity [6]. OS benefit was modest, with patients in the trifluridine/tipiracil arm living 1.8 months longer than those in the placebo arm. Median OS was 5.3 months in the placebo arm and 7.1 months in the trifluridine/tipiracil arm [6]. This was consistent with a prolongation of median OS from 6.6 months with placebo to 9 months with trifluridine/tipiracil that was shown in a smaller randomized phase 2 trial (J-003) from Japan [7]. Objective responses (all partial) were observed in 1.6% of patients receiving trifluridine/tipiracil in the RECOURSE trial, and 42.4% of patients had stable disease for at least 6 weeks after randomization, with a disease control rate (DCR) of 44%. A phase 3 trial from Asia (the TERRA trial) showed a statistically significant but modest benefit for OS and PFS of trifluridine/tipiracil over placebo [8]. The mean OS was prolonged from 7.1 months in the placebo arm to 7.8 months in the trifluridine/tipiracil arm and the PFS was prolonged from 1.8 months in the placebo arm to 2 months in the trifluridine/tipiracil arm [8]. A meta-analysis of published series of metastatic colorectal cancer patients treated with trifluridine/tipiracil showed a pooled median OS of 6.6 months (95% confidence interval: 6.1–7.1 months) and a pooled median PFS of 2.2 months (95% confidence interval: 2.1–2.3 months) [9].

In addition, trifluridine/tipiracil has been approved for the treatment of another difficult-to-treat gastrointestinal cancer, gastric and gastroesophageal junction adenocarcinoma, in patients that had previously received two or more standard of care lines of treatment [10]. The approval in this case was based on the multinational TAGS trial (NCT02500043) that showed prolongation in OS, PFS and the time to deterioration of ECOG performance status with trifluridine/tipiracil compared to placebo [11]. Median OS was prolonged from 3.6 months in the placebo group to 5.7 months in the trifluridine/tipiracil arm. The 1-year OS was 21% with trifluridine/tipiracil and 13% with placebo. Response rate in the trifluridine/tipiracil arm was 4% and DCR was 44%. A multicenter phase 2 trial from Japan confirmed a low response rate of 3.4% and showed a somewhat higher DCR of 65.5% [12]. Median OS and PFS were 8.7 months and 2.9 months, respectively.

These trial results and clinical experience prove that most patients progress through treatment and derive less or no benefit from trifluridine/tipiracil, being only exposed to its toxicity. This paper examines putative predictive markers for determining subsets of patients that are more likely to benefit from the drug.

## 2. Trifluridine/Tipiracil Pharmacology and Mechanism of Action

**Figure 2:**
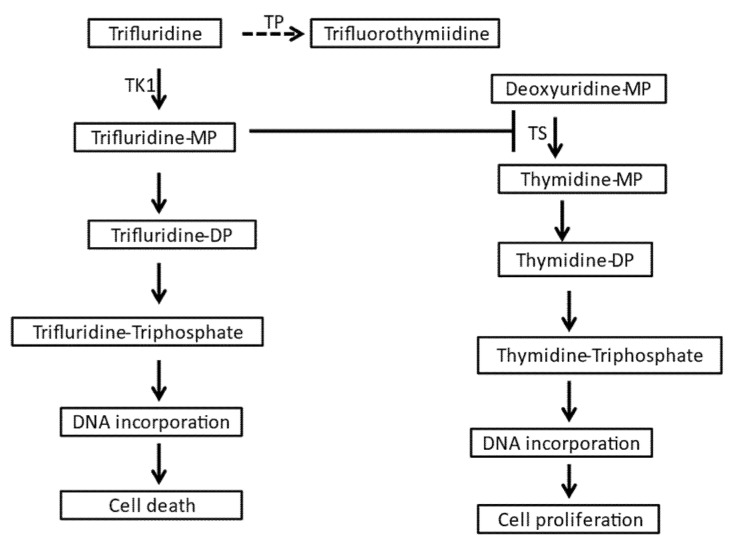
Pathways of trifluridine metabolism and action. DP: biphosphate, MP: monophosphate, TK1: thymidine kinase 1, TP: thymidine phosphorylase, TS: thymidine synthetase.

Trifluridine/tipiracil is available in tablets of fixed ratio of the two components containing 20 mg of trifluridine with 8.19 mg of tipiracil or 15 mg of trifluridine with 6.14 mg of tipiracil [5]. The approved dose is 35 mg/m^2^ of trifluridine twice daily on days 1 to 5 and days 8 to 12 of a 28 day cycle. Trifluridine is an antimetabolite that is phosphorylated by thymidine kinase 1 (TK1) and, after triphosphorylation, is incorporated into DNA, resulting in DNA damage and cell death [13,14]. In addition, the intermediate monophosphorylated form of trifluridine inhibits the enzyme thymidylate synthase (TS), interfering with DNA synthesis (Figure 2). DNA incorporation appears to be the main cytotoxic mechanism in the currently used clinical formulation of oral intermittent dosing [15]. In contrast, a better inhibition of thymidylate synthase is obtained with continuous schedules that are not currently used clinically [16]. Despite being water soluble, trifluridine is absorbed well in the intestine with about 60% of the drug becoming bioavailable in the circulation [17]. Trifluridine is transported inside intestinal epithelial cells through the sodium-dependent nucleoside transporter of the human concentrative nucleoside transporter family (hCNT), hCNT1 (also called SLC28A1) [18]. In addition, the human equilibrative nucleoside transporter family (hENT) transporters may play a role in trifluridine uptake in the intestine [19]. hENT1 (also called SLC29A1) and hENT2 transporters are responsible for the transfer of the drug from the basolateral side of the epithelium into the circulation [19]. After oral administration, trifluridine is catabolized on first pass in the liver by the enzyme thymidine phosphorylase (TP) to the inactive metabolite trifluorothymidine, which is then excreted by the kidneys [15]. Tipiracil is an inhibitor of TP that prevents first-pass catabolism of trifluridine, increasing its concentration and allowing for oral administration [10]. After reaching cancer cells, trifluridine is transported intracellularly by hENT1 to exert its antitumor effects [20]. Trifluridine persists inside tumor cells for at least 13 days following the last administration, while persistence is shorter in the bone marrow [21].

Tipiracil, the second component of TAS-102, besides being critical in avoidance of trifluridine first-pass liver degradation, has antitumor activity through the inhibition of angiogenesis. This activity relates to the inhibition of TP, which is a pro-angiogenic factor. TP (also called Platelet Derived Endothelial Cell Growth Factor) plays roles in wound healing and tumor angiogenesis and is upregulated in colorectal cancer [22]. Thus, trifluridine/tipiracil may be particularly suitable as a partner of therapy with other angiogenesis targeting agents. Inhibition of angiogenesis with the use of anti-VEGF monoclonal antibody bevacizumab in combination with trifluridine/tipiracil improves the outcomes of metastatic colorectal cancer patients who are not candidates for intensive therapy compared with the combination of capecitabine and bevacizumab [23]. Other studies have shown promising results with combinations of trifluridine/tipiracil and anti-angiogenic drugs in both colorectal and gastric cancers [24,25].

## 3. Biomarkers from Preclinical Models

In an in vitro study using H630 colorectal cancer cells that had acquired resistance to trifluridine by continuous or intermittent exposure to the drug, key molecular factors associated with resistance were suggested to be a decreased activity of activating enzyme TK1 and decreased levels of the equilibrative nucleoside transporter hENT [26]. In contrast, concentrative nucleoside transporters did not seem to be associated with trifluridine resistance. In addition, expression of serum phospholipase A2 (sPLA2) was increased in resistant cells, which was partially reversed by a sPLA2 inhibitor. These data suggest that, besides proteins directly involved in trifluridine uptake and activation, lipid metabolism perturbation contributes to resistance in this model.

The activity of TK1 was examined in a study of colorectal cancer cell line HCT-116, which was knocked down for TK1 through CRISPR/Cas9 [27]. Compared with parental cells, cells without TK1 expression developed resistance to trifluridine/tipiracil. In contrast, innate expression of TK1 in different colorectal cancer cell lines showed no correlation with trifluridine/tipiracil sensitivity, a fact that the authors attribute to different genetic backgrounds, which introduce additional and diverse molecular defects that could affect sensitivity [27]. Moreover, acquired resistance to trifluridine in the colorectal cancer cell line DLD1 is associated with loss of function of TK1 due to a point mutation in its gene, and resistance ensues in these cells through knockout of the gene through CRISPR/Cas9 [28]. Restoration of TK1 expression restores trifluridine sensitivity. In a human xenograft model in mice, TK1 levels in the xenografts as well as the ratio of TK1 to TP were associated with trifluridine/tipiracil sensitivity [29].

Consistent with a lesser role of TS inhibition for the antitumor effect of trifluridine/tipiracil, colorectal cancer cells with high TS expression were more sensitive to trifluridine/tipiracil compared to 5-FU [30]. The intracellular expression of TP in colorectal cancer cells plays a minor role in trifluridine efficacy due to prompt activation of the drug to the monophosphorylated form by TK1 [31]. This fact confirms that the main effects of trifluridine potentiation by tipiracil occur via the inhibition of liver first-passage metabolism.

To examine whether the mismatch repair (MMR) status of colorectal cancer cells affects sensitivity to trifluridine/tipiracil, a study used the MMR deficient cell line HCT116, which is defective for the mismatch repair protein MLH1, and a derivative cell line, HCT116 + ch3, with restoration of MLH1 expression [32]. No difference in survival between HCT116 and HCT116 + ch3 cells was observed after exposure to the drug.

## 4. Clinical Biomarkers

Investigators of the RECOURSE trial performed a subgroup analysis to examine clinical factors potentially associated with trifluridine/tipiracil benefit on OS and PFS [6]. These included time from first diagnosis of metastatic disease (shorter or longer than 18 months), area of recruitment (Japan or Western countries), sex, age above 65, ECOG performance status, primary colon or rectal disease, prior use of regorafenib and number of metastatic sites (one–two versus three or more). None of these factors showed significant influence on the benefit obtained by trifluridine/tipiracil treatment [6]. Patients who had been treated with only two prior regimens seem to derive no OS benefit from trifluridine/tipiracil treatment (hazard ratio versus placebo 1.05, 95% confidence interval: 0.68–1.63), while patients who had received three prior regimens or four and more prior regiments derived incremental benefits (hazard ratio versus placebo 0.74, 95% confidence interval: 0.54–1.08 and 0.59, 95% confidence interval: 0.47–0.73, respectively). This differential benefit was not observed in the TAGS trial of gastric and gastroesophageal adenocarcinoma where a similar benefit was evident in patients who had received two, three, or more than three prior regimens with hazard ratios for OS versus placebo between 0.68 and 0.73 [11]. In addition, similar to the RECOURSE trial, no other significant differences were observed in the sub-group analysis of the TAGS trial. Notably, patients with gastric and gastroesophageal junction primaries had similar benefit from trifluridine/tipiracil (hazard ratio versus placebo 0.67, 95% confidence interval: 0.52–087 and 0.75, 95% confidence interval: 0.50–1.11, respectively) [11,33]. Moreover, patients derived similar benefits from trifluridine/tipiracil independently of whether they had previously undergone gastrectomy [34].

In a cohort of 379 metastatic colorectal cancer patients who received trifluridine/tipiracil mostly as a third or later-line treatment in the Spanish multicenter ROS study, the median OS was 7.9 months [35]. Patients with a maximum of two metastatic sites, without liver metastases, with alkaline phosphatase less than 300 IU and with a neutrophil-to-lymphocyte ratio of less than five had a significantly longer OS than patients with three or more metastatic sites, liver metastases, alkaline phosphatase levels above 300 IU and a neutrophil-to-lymphocyte ratio above five [35].

REGOTAS was a non-randomized, multicenter, retrospective evaluation of patients with metastatic colorectal cancer who received either trifluridine/tipiracil or regorafenib [36]. The study showed no difference for survival outcomes between the two drugs. Among factors examined for a putative association with greater benefit from either drug, the trifluridine/tipiracil benefit seemed to be more extensive for patients older than 65 years-old, while regorafenib was more beneficial in younger patients [36]. Age older than 65 years-old was also the only factor associated with benefit from trifluridine/tipiracil in prespecified sub-group analysis in the TERRA trial [8]. The hazard ratio for OS of the trifluridine/tipiracil group compared with the placebo group was 0.45 (95% confidence interval: 0.28–0.74) in patients 65 years-old and older, and it was 0.90 (95% confidence interval: 0.69–1.18, *p* = 0.026) in patients younger than 65 years-old. Primary tumor location (left versus right colon) was not associated with trifluridine/tipiracil or regorafenib benefit in the REGOTAS study [37]. In addition, there was no difference in the benefit of patients with primary colon versus rectal cancer in the sub-group analysis of TERRA trial [8].

## 5. Biomarkers from the Circulation

In a retrospective analysis from the RECOURSE trial, patients in the trifluridine/tipiracil arm who developed any grade neutropenia at the first or second cycle of treatment had a better OS and PFS than patients without neutropenia [38]. The median OS of patients with neutropenia was 9.3 months, and it was 4.4 months in patients without neutropenia at the first or second cycle of treatment. The benefit was even more prominent in patients with grade 3 and higher neutropenia compared with patients with grade 1 and 2 neutropenia. Patients without neutropenia had a median OS similar to the median OS of patients in the placebo arm. Median PFS was 3.5 months in patients with neutropenia and 1.8 months in patients without neutropenia in the first or second cycle of treatment [38]. Pharmacokinetic studies in a subset of patients from the trifluridine/tipiracil arm of RECOURSE showed that development of neutropenia was associated with higher exposure to trifluridine. Grade 3 and 4 neutropenia at the first trifluridine/tipiracil cycle was also a significant predictor of PFS compared with patients with lower grade neutropenia in a series of 95 metastatic colorectal cancer patients from Japan [39]. OS was also longer in patients with grade 3 and 4 neutropenia but was just short of reaching statistical significance (log rank *p* = 0.08). In another Japanese series of 149 patients treated with trifluridine/tipiracil, patients with grade 2 or higher neutropenia at 1 month of treatment had a better PFS and OS than patients with grade 1 or no neutropenia [40]. Grade 2 and higher neutropenia and higher CEA levels were independent prognostic factors for OS. Patients in the ROS study who had trifluridine/tipiracil dose reductions, presumably due to neutropenia, had a longer OS than that of patients who did not need a dose reduction [35].

Grade 3 and 4 neutropenia at the first cycle was also associated with a longer PFS in metastatic colorectal cancer patients who received the combination of trifluridine/tipiracil with bevacizumab [41].

As mentioned above, two peripheral blood laboratory values, the neutrophil-to-lymphocyte ratio and alkaline phosphatase, together with two clinical factors, the number of metastatic sites and the presence of liver metastases, were prognostic for trifluridine/tipiracil efficacy in the ROS cohort [35]. In another small retrospective series of 33 metastatic colorectal cancers treated with trifluridine/tipiracil, patients with neutrophil-to-lymphocyte ratios above five before start of treatment had significantly worse OS and PFS rates than those of their counterparts with neutrophil-to-lymphocyte ratios below five [42]. However, due to lack of a comparative group that did not receive the drug, it is impossible to confirm that the neutrophil-to-lymphocyte ratio is a genuine predictive value of response to trifluridine/tipiracil and not merely a prognostic factor of worse outcomes in metastatic colorectal cancer, as previously confirmed [43]. A study from the REGOTAS cohort showed the prognostic value of the modified prognostic Glasgow score (mGPS, an index attributing points for hypoalbuminemia and elevated CRP) for both patients treated with trifluridine/tipiracil and treated with regorafenib [44]. Patients treated with trifluridine/tipiracil and an mGPS of 0 had an OS of 9.6 months, when mGPS was 1 the OS dropped to 5.3 months, and OS was 4.2 months when mGPS was 2. Since this study was predictive of outcomes in both patients receiving trifluridine/tipiracil and patients receiving regorafenib, it is impossible to differentiate whether mGPS is a predictive factor for both treatments, or if it is a prognostic factor for all patients with metastatic colorectal cancer. The same investigators also proposed a prognostic index consisting of AST, CRP, tumor marker Ca19-9 and ECOG performance status as predictive of OS in patients receiving trifluridine/tipiracil [45]. Patients with AST above 40 IU/L, CRP above 1 mg/dL, Ca19-9 above 37 U/mL and ECOG performance status of 1 or 2 had a median OS of 2.8 months (95% confidence interval: 2.0–3.5 months), while patients with none of these factors had a median OS of 15.4 months (95% confidence interval: 9.7–21.2 months). Patients with one to three factors had an intermediate median OS of 7.5 months (95% confidence interval: 6.6–8.3 months) [45].

The ratio of lymphocytes to monocytes was explored as a predictive biomarker of response in a phase 2 trial of trifluridine/tipiracil plus bevacizumab as a third-line treatment in metastatic colorectal cancer [46]. Patients with a high lymphocyte-to-monocyte ratio had a longer disease control rate than patients with a lower lymphocyte-to-monocyte ratio (87.5% vs. 43.8%).

## 6. Biomarkers from the Tumor and Genetic Polymorphisms

*KRAS* is an oncogene involved in colorectal cancer pathogenesis, and it is mutated in about one-third of colorectal cancers. In the RECOURSE trial, both patients with and without *KRAS* mutations benefited from trifluridine/tipiracil treatment, although the benefit was more pronounced in patients with *KRAS* wildtype disease (hazard ratio for OS versus placebo 0.58, 95% confidence interval: 0.45–0.74) than in patients with KRAS mutant disease (hazard ratio for OS versus placebo 0.80, 95% confidence interval: 0.63–1.02) [6].

TK1 expression levels in the tumor were evaluated by immunohistochemistry in a pooled analysis of 329 patients participating in the J-003 trial and patients recruited from Japan in the RECOURSE trial [47]. Expression of TK1 was categorized as high with a cut-off of 15% based on the percentage of tumor cells staining positive for TK1 with a score of 2+ or 3+. The OS benefit of trifluridine/tipiracil over placebo was higher (hazard ratio 0.65, 95% confidence interval: 0.46–0.93) in patients with high TK1 expression compared with patients with low TK1 expression who derived less benefit from the drug (hazard ratio 0.88, 95% confidence interval: 0.63–1.23). However, the benefit regarding PFS and DCR was balanced in both the TK1 high and low groups [47].

A nucleotide transporter hENT1 (SLC29A1) polymorphism (rs760370A > G) was associated with PFS and OS in patients with colorectal cancer treated with trifluridine/tipiracil [48]. Patients with at least one polymorphic G allele had a longer PFS and OS than those of patients homozygous for the A allele. Median OS was 8.7 months in heterozygous patients or patients with the GG phenotype and 5.3 months in patients with the AA phenotype. In contrast, the prognosis of patients treated with regorafenib was not affected by the hENT1 polymorphism [48]. Two additional polymorphisms in enzymes involved in elimination of tipiracil, MATE1 and OCT2 may add prognostic information when supplementing the evaluation of hENT1 rs760370A > G polymorphism [48].

## 7. Perspective and Conclusions

Clinical and molecular predictive biomarkers for trifluridine/tipiracil response in gastrointestinal cancers would be valuable for improving the use of the drug in the clinic. Such biomarkers have been investigated but have not been incorporated in clinical practice yet due to several hurdles. The overview of the most promising clinical biomarkers in the previous sections discloses that such predictors include early neutropenia during treatment and composite biomarkers consisting of laboratory and clinical parameters. However, the retrospective nature of these data, as well as the variations of the proposed combinations, impedes a systematic development and precludes their advancement to practice. In addition, neutropenia during treatment, which appears to be a consistent predictor in several reports, is a suboptimal biomarker due to availability only during therapy. This is a significant disadvantage, especially given the short PFS of the treatment.

Regarding molecular markers, enzymes involved in uptake and activation of trifluridine, including transporter hENT1 and TK1, could be of interest as predictive markers. *KRAS* mutations are common in colorectal cancers and are present in about one-third of patients. They have been used clinically to determine a lack of response to targeted anti-EGFR therapies. Subgroup analysis from the RECOURSE trial suggests that presence of *KRAS* mutations may also be a marker of lack of benefit from trifluridine/tipiracil. *KRAS* mutations lead to activation of the KRAS/BRAF/MEK/ERK pathway with downstream activation of transcription factors mediating cellular proliferation [49]. TK1 is an enzyme induced when cells proliferate and could be the link between *KRAS* mutations and resistance to trifluridine/tipiracil. KRAS and downstream pathway activation are associated with resistance to treatments, including other antimetabolites, such as gemcitabine [50]. *KRAS* mutations as well as putative roles of other common molecular defects of colorectal cancer as biomarkers deserve further investigation.

Prospective investigations or integration of biomarker evaluation in prospective trials will be needed to advance the field and move both molecular and clinical biomarkers of trifluridine/tipiracil efficacy in clinical practice. A pragmatic design for a prospective trial of neutropenia as a biomarker could include a randomization of patients with no grade 2 or higher neutropenia before cycle 2 to dose escalation versus continuation of the standard dose of the drug. Development of biomarkers from tumor biopsies such as TK1 expression will also require an analytical step of standardization of immunohistochemistry and determination of optimal cut-offs. An optimized determination of patients more likely to respond will improve the care of patients receiving the drug and may also help with further development of combination therapies of trifluridine/tipiracil.
